# Effects of Climate, Sun Exposure, and Dietary Intake on Vitamin D Concentrations in Pregnant Women: A Population-Based Study

**DOI:** 10.3390/nu15051182

**Published:** 2023-02-27

**Authors:** Ya-Li Huang, Thu T. M. Pham, Yi-Chun Chen, Jung-Su Chang, Jane C.-J. Chao, Chyi-Huey Bai

**Affiliations:** 1 Department of Public Health, School of Medicine, College of Medicine, Taipei Medical University, Taipei 110-31, Taiwan; 2 School of Public Health, College of Public Health, Taipei Medical University, Taipei 110-31, Taiwan; 3 Faculty of Public Health, Hai Phong University of Medicine and Pharmacy, Hai Phong 042-12, Vietnam; 4 School of Nutrition and Health Sciences, Taipei Medical University, Taipei 110-31, Taiwan; 5 Nutrition Research Center, Taipei Medical University Hospital, Taipei 110-31, Taiwan; 6 Graduate Institute of Metabolism and Obesity Sciences, College of Nutrition, Taipei Medical University, Taipei 110-31, Taiwan; 7 Chinese Taipei Society for the Study of Obesity, Taipei 110-31, Taiwan; 8 Master Program in Global Health and Development, College of Public Health, Taipei Medical University, Taipei 110-31, Taiwan

**Keywords:** 25-hydroxyvitamin D [25(OH)D] concentration, diet, pregnant women, sunlight, Taiwan, vitamin D deficiency

## Abstract

Background: Vitamin D deficiency (VDD) is a global micronutrient issue that commonly occurs in pregnant women, leading to adverse health outcomes. We examined the role of sunlight-related factors and dietary vitamin D intake on vitamin D concentrations among pregnant women in different climate zones. Methods: We conducted a nationwide cross-sectional survey in Taiwan between June 2017 and February 2019. The data of 1502 pregnant women were collected, including sociodemographic information and characteristics related to pregnancy, diet, and sun exposure. Serum 25-hydroxyvitamin D concentrations were measured, and VDD was assessed as a concentration of less than 20 ng/mL. Logistic regression analyses were used to explore the factors associated with VDD. Furthermore, the area under the receiver operating characteristic (AUROC) curve was used to analyze the contribution of sunlight-related factors and dietary vitamin D intake to vitamin D status stratified by climate zones. Results: The prevalence of VDD was 30.1% and was the highest in the north. Sufficient intake of red meat (odds ratio (OR): 0.50, 95% confidence interval (CI): 0.32–0.75; *p* = 0.002), vitamin D and/or calcium supplements (OR: 0.51, 95% CI: 0.39–0.66; *p* < 0.001), sun exposure (OR: 0.75, 95% CI: 0.57–0.98; *p* = 0.034), and blood draw during sunny months (OR: 0.59, 95% CI: 0.46–0.77; *p* < 0.001) were associated with a lower likelihood of VDD. Additionally, in northern Taiwan, which is characterized by a subtropical climate, dietary vitamin D intake (AUROC: 0.580, 95% CI: 0.528–0.633) had a greater influence on vitamin D status than did sunlight-related factors (AUROC: 0.536, 95% CI: 0.508–0.589) with a *z* value = 51.98, *p* < 0.001. By contrast, sunlight-related factors (AUROC: 0.659, 95% CI: 0.618–0.700) were more important than dietary vitamin D intake (AUROC: 0.617, 95% CI, 0.575–0.660) among women living in tropical areas of Taiwan (*z* value = 54.02, *p* < 0.001). Conclusions: Dietary vitamin D intake was essential to alleviate VDD in the tropical region, whereas sunlight-related factors played a greater role in subtropical areas. Safe sunlight exposure and adequate dietary vitamin D intake should be promoted appropriately as a strategic healthcare program.

## 1. Introduction

Vitamin D deficiency (VDD) has become an urgent micronutrient issue globally [1] because of its high prevalence [2], and it has become a potential cause of non-communicable [3,4] and infectious [5,6] diseases. Although VDD has been addressed as a global public health problem in all age groups, the population-representative data regarding vitamin D were limited to several risky groups [7]. Pregnant women are a vulnerable population affected by VDD [1], which can lead to adverse pregnancy outcomes [8,9]. Moreover, VDD may result in health disparities [10], which leads to the increment of stillbirths and pregnancy-related deaths [11]. Hence, improving vitamin D status is necessary to upgrade the reproductive health and well-being of mothers and their infants.

The major factors for VDD are sun exposure and dietary vitamin D intake [12]. However, obtaining vitamin D through sun exposure can be inefficient or unsafe because of the skin cancer risk from ultraviolet radiation [13]. Additionally, the dermal synthesis of vitamin D was suggested to be influenced in different climate zones using an in vitro model [14]. The adequate achievement of vitamin D intake from diet alone is hard [15]. Therefore, vitamin D supplementation is a crucial nutritional priority recommended by many physicians to achieve optimal serum concentration [16] that could prevent short and long-term maternal and infant health complications [17].

Vitamin D status has been explored in the literature. However, population-based research on pregnant women in East Asia is still limited. To our best knowledge, relevant information regarding the potential effect of the climatic zone has not been explored. Taiwan is an East Asian island characterized by two climatic zones [18]. Based on this unique advantage, Taiwan has the opportunity to assess whether sunlight-related factors and dietary vitamin intake contribute differently to vitamin D levels among people living in different parts of the country. Exploring the prevalence of VDD and its potential risk factors among pregnant women in Taiwan is an important task to address the research gap and for future policy planning. This study aimed to assess the determinants of VDD and to examine the contribution of sunlight-related factors and dietary vitamin D intake to vitamin D status in different regions of Taiwan using a nationally representative survey.

## 2. Materials and Methods

### Study Population

**Figure 1:**
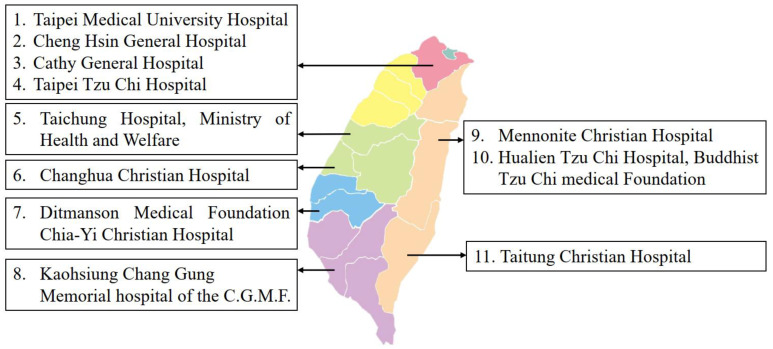
The distribution of eleven selected hospitals across Taiwan.

A national cross-sectional nutritional survey of pregnant women was conducted from June 2017 to February 2019 across Taiwan. A multiple-stage cluster sampling approach was used, including (1) the selection of eight layers according to geographical location (northern, central, southern, and eastern Taiwan) and (2) the random selection of hospitals (large and small sizes) from the list based on the number of women availing pregnancy-related services per year and the probability proportional to size in each layer and (3) the whole selection of participants arriving in the selected hospitals for antenatal examination with the expectation of 150–300 women from one or two hospitals in each layer enrolled based on the potential number of annual outpatients in each hospital [19]. The distribution of eleven selected hospitals across Taiwan was in Figure 1.

We calculated a sample size of 1062 based on 200,000 deliveries by pregnant women during the study period, with a 3% margin of error and a 95% confidence interval (CI). We recruited participants aged ≥15 years who were legal residents of Taiwan and who underwent antenatal examinations at the selected hospitals. A satisfactory sample of 1502 pregnant women was included in the final analysis after the exclusion of nonsingleton pregnancies, participants unable to understand and speak Mandarin, and incomplete questionnaires. All participants provided written informed consent before taking the survey.

## 3. Data Collection

During study periods, all pregnant women making an antenatal visit were enrolled consecutively. At recruitment, collection of questionnaires, physical examination and blood sample were performed. Information was obtained from standardized face-to-face interviews by trained interviewers using the structured questionnaires. Variables regarding participants’ sociodemographic status, histories of diseases before and during pregnancy, pregnancy-related factors, and intake histories of prenatal and natal dietary supplements were collected by the self-reported baseline questionnaire. The dosage of supplements during pregnancy was asked and recorded in brand, exact dosage and frequency per week. Food frequency questionnaires was also used to record the intake frequency during past 3 months in 66 items of foods including egg, milk, meat, fish and vegetables. After interview of questionnaires, a 24 h dietary recall was recorded by trained dietitians. Food models were used to assist participants in recalling the food portion sizes and details of the dietary information. Then, we estimated participants’ energy intake and nutrient intake from foods. The intakes of several nutrients (e.g., vitamin D) were labeled the sources of foods or supplements respectively. We used the online software Cofit Pro (Cofit Health Care, Taipei, Taiwan) to analyze participants’ nutrient intake using the 2015 version of the Taiwan Food and Nutrient Database.

At the time of recruitment, pre-pregnancy body weight was self-reported by pregnant women, and their current body height and weight were measured. Blood samples were drawn, centrifuged, then froze (−80 °C) and analyzed in batches. 

### 3.1. Sociodemographic and Pregnancy-Related Characteristics

Pregnant women were queried regarding their age (years); residential area; education level; household monthly income; religion; gravidity; parity; number of fetuses in the current pregnancy; gestational age; and body height (cm) and weight (kg) before pregnancy, which were used to calculate pre-pregnancy body mass index (BMI, kg/m^2^). Additional information related to pregnancy was extracted from the prenatal visit records of participants. The residence was categorized as living in Taiwan’s northern, central, southern, or eastern regions. 

### 3.2. Dietary Characteristics

Pregnant women were asked whether they consumed sufficient amounts of the four groups of the following food items: (1) dairy products (e.g., fresh milk, yogurt, cheese, cream cheese, and powdered milk); (2) eggs; (3) red meat (e.g., pork, beef, and mutton); and (4) nut fruits (e.g., stone fruit, nuts, pistachios, and almonds). Women also reported their frequency of using vitamin D and/or calcium supplements during pregnancy as “never”, “less than 1 day per week”, “2–5 days/week”, and “almost daily”. Then, this factor was recoded into two categories of usage, “yes” or “no”, due to the small sample size. The 24 h dietary intake was recorded to assess the intake of total energy (kcal), raw protein (g), raw fat (g), total carbohydrates (g), and vitamin D content (mg) and the use of vitamin supplements. The percentages of calories from protein, fat, and carbohydrates were also calculated [19].

The dosages of supplements were calculated if participants provided the exact dosage. However, these parameters were frequently missing, as were the brands and models of vitamins. Therefore, in the present study, we only analyzed the usage frequency of vitamin D-only or D-based supplements.

### 3.3. Sunshine-Related Factors

Sun exposure was estimated using the question, “Were you exposed to outdoor sunlight last month?” and the answers were categorized as “no” if exposed to sunlight for less than 10 min per day and “yes” if exposed to sunlight for more than 10 min per day. The seasons of blood draw were categorized according to the month of blood sample collection, as follows: sunny months (June to November) and rainy months (December to May) established according to the rainfall report of the Central Weather Bureau, Taiwan. Participants also reported whether they had to stay indoors (e.g., bedridden) for any reason during their pregnancy (“yes” or “no” response) and the number of methods used for sun protection (e.g., sunscreen, parasols, hats and outerwear with UV-block) and how often they are used. 

### 3.4. Vitamin D Deficiency Assessment

As 25-hydroxyvitamin D [25(OH)D] has the long half-life (15 days) and relative stability of concentration in the blood [20], the circulating 25(OH)D is the useful biomarker of vitamin D in the human body [21]. The plasma 25-hydroxyvitamin D [25(OH)D] concentration was measured using an electrochemiluminescence immunoassay, as described previously [19]. Although there is no consensus in the definition of the suboptimal vitamin D level, VDD was defined as a 25(OH)D level of <20 ng/mL, which is a common threshold for people in at-risk groups, including pregnant women [22,23,24]. The cutoff point of less than 20 ng/mL was also recommended for use for VDD by Institution of Medicine, Academy of Medicine and American Academy of Pediatrics.

## 4. Ethical Consideration

This study was funded by the Health Promotion Administration, Ministry of Health and Welfare in Taiwan (C1050912) and was approved by the institutional review board of the government and selected hospitals (IRB number: N201707039).

## 5. Statistical Analysis

First, descriptive analysis was performed to explore the distribution of independent variables. We performed chi-square tests (for categorical variables) and *t* tests or Mann–Whitney tests (for continuous variables) to compare the distribution of independent variables between pregnant women with and without VDD. Second, logistic regression analysis was used to determine the factors associated with VDD. Two models were constructed. Model 1 comprised variables associated with VDD that had *p* < 0.1 in bivariate analysis, including age, residential area, parity, gestational age, pre-pregnancy BMI, egg intake, red meat intake, fat, vitamin D content, vitamin supplements, sun exposure, remaining indoors during pregnancy, and the season of blood draw. Gravidity and carbohydrate intake were removed from model 1 because they were highly correlated with parity (*rho* = 0.82) and fat intake (*rho* = −0.89), respectively (Table S1). Model 2 comprised factors associated with VDD that had *p* < 0.1 in model 1, including age, residential area, gestational age, red meat intake, vitamin D content, vitamin supplements, sun exposure, remaining indoors during pregnancy, and the season of blood draw. Odds ratios (ORs) and 95% CIs were reported, and *p* < 0.05 was considered statistically significant.

Further sensitivity analysis was performed and stratified by residential area (north vs. south and other regions) to examine the contribution of modifiable factors to vitamin D status. Two models were constructed for each layer, including one model adjusted for sunlight-related factors (season of blood draw and sun exposure) and one model adjusted for dietary vitamin D intake (red meat and supplements). The area under the receiver operating characteristic (AUROC) curve was computed to compare the models. It is favored due to the characteristics of invariant and independent from the prevalence of the condition. All analyses were performed using R software (version 4.1.3; R Foundation for Statistical Computing, Vienna, Austria). 

## 6. Results

### 6.1. Characteristics of Study Participants

The data contained several missing values, but the distribution of variables before and after removing the missing information was the same. Therefore, the entire data of the 1502 pregnant women were used for analysis. Overall, the mean 25(OH)D concentration was 25.5 ± 8.9 ng/mL, and the prevalence of VDD was 30.1% (weighted). Compared with women without VDD, those with VDD were younger (*p* = 0.017); lived in the north (*p* < 0.001); had uniparity (*p* = 0.01); were in the first trimester of gestation (*p* < 0.001); consumed high quantities of carbohydrates (*p* = 0.013) but insufficient eggs (*p* = 0.034), red meat (*p* < 0.001), fat (*p* = 0.023), and vitamin D and/or calcium supplements (*p* < 0.001); had little sun exposure (*p* = 0.001); remained indoors during pregnancy (*p* = 0.018); and had blood drawn during the rainy months (*p* = 0.004). These data are displayed in (Table 1).

### 6.2. Associated Factors of Vitamin D Deficiency

As displayed in Table 2, the likelihood of VDD was significantly lower in pregnant women who were older (OR: 0.95, *p* < 0.001); lived in central (OR: 0.66, *p* = 0.010), southern, or eastern Taiwan (OR: 0.20, *p* < 0.001) or in the eastern and outlying islands (OR: 0.33, *p* < 0.001); were in the second trimester (OR: 0.72, *p* = 0.046) or the third trimester (OR: 0.60, *p* = 0.002); consumed sufficient red meat (OR: 0.50, *p* = 0.002); took vitamin D and/or calcium supplements (OR: 0.51, *p* < 0.001); received sun exposure (OR: 0.75, *p* = 0.034); and had blood drawn during the sunny months (OR: 0.59, *p* < 0.001).

**Figure 2:**
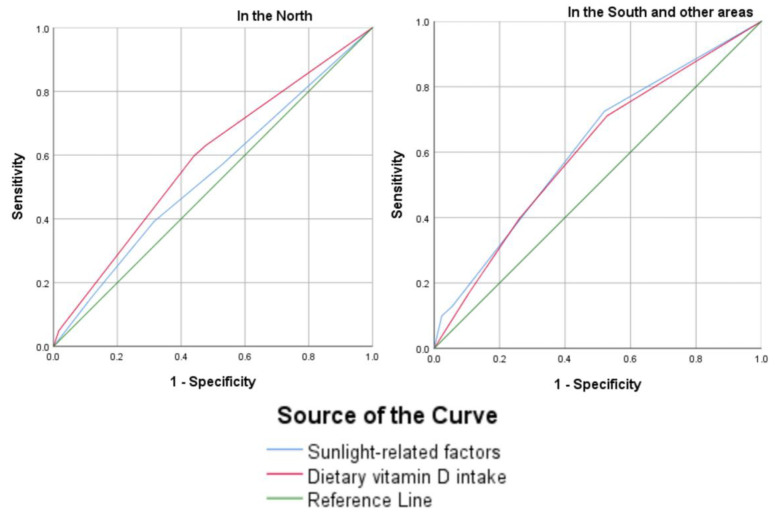
Contribution of sunlight-related factors and dietary vitamin D intake to vitamin D status in different regions of Taiwan.

In the sensitivity analysis, among participants living in northern Taiwan, dietary vitamin D intake (AUROC: 0.580, 95% CI: 0.528–0.633) had a greater influence on vitamin D status than did sunlight-related factors (AUROC: 0.536, 95% CI: 0.508–0.589). By contrast, among participants living in the south and other parts of Taiwan, sunlight-related factors (AUROC: 0.659, 95% CI: 0.618–0.700) were more influential than dietary vitamin D intake (AUROC: 0.617, 95% CI: 0.575–0.660). The differences in regional models were significant, with *z* value = 51.98, *p* < 0.001 for northern Taiwan and *z* value = 54.02, *p* < 0.001 for the remaining regions. These results are visualized in Figure 2. 

## 7. Discussion

In the present study, the prevalence of 25(OH)D level < 20 ng/mL among pregnant women in Taiwan was 30.1% (weighted). The determinants of VDD included age, gestational age, red meat intake, vitamin D and/or calcium supplements, residential area, sun exposure, and the season of blood draw.

The occurrence of VDD [25(OH)D < 20 ng/mL] is common in pregnant women, although the rates vary in different Asian countries, ranging from 7% to 40.7% [25,26]. The present study found that VDD occurred more frequently in pregnant women living in northern Taiwan than in those living in southern Taiwan. A nationwide report on VDD among older adults (a risk group of VDD) had similar findings, reporting that VDD occurrence was higher in the north than in the south [27]. This phenomenon has several possible explanations. First, northern Taiwan has a higher latitude than other regions [28], and vitamin D status decrease with increasing latitudes [29]. Second, northern Taiwan has a humid subtropical climate, and sunlight may be of lower intensity than that in southern Taiwan and other regions characterized by a tropical monsoon climate. The association between age and VDD was found in the previous studies with the controversial findings. The former authors showed that age over thirty was the risk factor for VDD among pregnant women [26]. However, the current study indicated that younger age was a contributing factor for VDD, which was in line with other studies [30,31]. Our findings could be due to the habits of avoiding sunlight among almost youngers that they were likely to apply sun protection (e.g., using sunscreen, wearing long-sleeved clothes, preferring indoor activities). Thus, our findings indicate that it is worth planning VDD prevention, such as educating health literacy related to VDD and lifestyle changes in younger women, and such methods should be promoted integrating with efficient intervention strategies. 

Regarding the impact of gestational age on maternal VDD, the findings are inconsistent across studies. Although several studies have reported that vitamin D status decreased during advanced gestation [32], our results are in line with those of studies reporting that the likelihood of VDD was reduced during the second and third trimesters. For example, Perreault et al. indicated that serum 25(OH)D concentrations were significantly greater in the last trimester compared to the first trimester [33]. Similarly, Savard et al. found that serum 25(OH)D levels significantly increased across trimesters [34]. In addition, Shen et al. noted a positive relationship between the increased vitamin D concentration and later gestational week [35].

It has been well established that sunlight is the main source of vitamin D. Hence, sun exposure and the summer season are the most important contributing factors to the vitamin D concentration. Nevertheless, if sun exposure cannot provide sufficient vitamin D because of factors such as sunlight intensity, time of exposure, and application of sun protection, the vitamin D status in the human body can be adjusted through nutrition and dietary intake. In the literature, the natural vitamin D content in foodstuffs is usually limited to vitamin D3 from animal products [36]. Our findings indicated that the consumption of red meat was associated with lower VDD rates. Moreover, the present study demonstrated that vitamin D and/or calcium supplements could reduce the likelihood of VDD.

In our sensitivity analysis, the effects of sunlight-related factors and dietary vitamin D intake on 25(OH)D levels varied by region. In northern Taiwan, dietary vitamin D intake was more important than sunlight-related factors for improving maternal vitamin D status; however, sunlight-related factors were the main sources of vitamin D for pregnant women living in the south and other parts of Taiwan, and vitamin D intake played a minor role. These variations in effectiveness corresponded to the variations in climate across Taiwan. These findings can assist health policymakers in designing regional strategies for the prevention of prenatal VDD. 

To date, suboptimal vitamin D levels is mostly indicated for bone health but remain controversial across populations and countries. For some investigators, deficiency was defined as specific to bone; however, insufficiency was defined relating to other health outcomes. For others, deficiency covered diseased population and insufficiency covered at-risk population. One of the most commonly used definitions comes from the Endocrine Society Clinical Practice Guidelines [24]; vitamin D deficiency was defined as 25(OH)D values below 20 ng/mL (50 nmol/L), and vitamin D insufficiency was defined as 25(OH)D of 21–29 ng/mL (52.5–72.5 nmol/L). This guideline was accepted and used widely by the International Osteoporosis Foundation, American Association for Clinical Endocrinologists, Institute of Medicine, American Academy of Pediatrics, and government of Australia, New Zealand, Germany, Austria and Switzerland as well as in Taiwan. In any case, cut point is very important when looking at the results in 25(OH)D level. 

Particularly in older adults, having a higher BMI or body fat percentage are significant subject-specific characteristics that negatively affect vitamin D metabolism [37]. Normal-weight women reached the higher 25(OH)d level after vitamin D supplementation faster than women with obesity [38]. However, in pregnant women, the association between BMI and VDD was not consistent across the studies. While several studies showed that high BMI was associated with VDD, others showed that BMI was not statistically significantly associated with VDD [39,40]. Obesity is strongly associated with insufficient dietary vitamin D intake and low sun exposure. Pre-pregnancy obesity predicts poor vitamin D status in mothers [41]. In our study, pre-pregnancy BMI (as a continuous variable) was significantly different in two groups of VDD and non-VDD, but in logistic regression, after adjusting for confounders, pre-pregnancy BMI was not significantly associated with VDD. The findings for BMI (as a categorical variable) were also insignificant in multiple logistic regression. Obesity is not associated with 25(OH)D levels in our study. 

The present study is the first national report on vitamin D status among pregnant women in Taiwan. Our findings demonstrated specific differences in the effects of sunlight-related factors and vitamin D intake on vitamin D concentrations in distinct regions of Taiwan. However, several limitations should be considered. First, because this was a cross-sectional study, we can only note associations; we cannot determine the causal relationship. Second, several factors influencing vitamin D status were not assessed in our study, such as occupation and the brand and dose of supplements. Third, we used a self-report questionnaire, which may introduce assessment bias because of subjective responses. Fourth, although the present study highlights the critical role of dietary vitamin D intake, the data on nutrient quantitation per serving are unavailable.

## 8. Conclusions

VDD was prevalent in pregnant women in Taiwan. On the basis of our findings, we recommend the promotion of a robust health policy regarding safe sunlight exposure and effective dietary vitamin D intake, with adjustments according to the characteristics of various climate zones. In doing so, clinicians can enhance maternal vitamin D status, reduce the VDD-induced burden, and improve health and well-being.

## Figures and Tables

**Table 1:** Characteristics of study participants according to vitamin D status (*n* = 1502).

Variables	Total	Non-VDD (1095, 72.9%)	VDD (407, 27.1%)	*p*
	*n* (%)	*n* (%)	*n* (%)	
Maternal age (years) (mean ± SD)	32.5 ± 4.8	32.7 ± 4.8	32.1 ± 4.8	0.017
Residential area				<0.001
North	501 (33.4)	312 (28.5)	189 (46.4)	
Central	371 (24.7)	260 (23.7)	111 (27.3)	
South and east	291 (19.4)	254 (23.2)	37 (9.1)	
Eastern and outlying islands	339 (22.6)	269 (24.6)	70 (17.2)	
Education level *				0.291
High school and below	237 (15.9)	182 (16.8)	55 (13.5)	
College, university	1025 (68.7)	740 (68.1)	285 (70.0)	
Postgraduate studies	231 (15.5)	164 (15.1)	67 (16.5)	
Household monthly income				0.465
Less than NT$30,000	212 (14.4)	162 (15.1)	50 (12.5)	
NT$30,000–59,999	634 (43.0)	464 (43.2)	170 (42.4)	
NT$60,000–99,999	443 (30.1)	318 (29.6)	125 (31.2)	
More than NT$100,000	185 (12.6)	129 (12.0)	56 (14.0)	
Religion				0.242
None	689 (45.9)	488 (44.6)	201 (49.4)	
Buddhism	281 (18.7)	205 (18.7)	76 (18.7)	
Taoism	345 (23.0)	265 (24.2)	80 (19.7)	
Other (Yiguandao, Christian, Catholic, Muslim)	187 (12.5)	137 (12.5)	50 (12.3)	
Gravidity *				0.061
1	694 (46.3)	487 (44.6)	207 (51.0)	
2	498 (33.2)	366 (33.6)	132 (32.5)	
3	199 (13.3)	158 (14.5)	41 (10.1)	
≥4	107 (7.1)	81 (7.4)	26 (6.4)	
The ordinal of current pregnancy (parity) *				0.010
1st child	824 (55.0)	577 (52.9)	247 (60.7)	
2nd child	527 (35.2)	395 (36.2)	132 (32.4)	
≥3rd child	146 (9.8)	118 (10.8)	28 (6.9)	
Number of fetuses in this pregnancy				0.972
≥2	33 (2.2)	24 (2.2)	9 (2.2)	
Gestational age				<0.001
1st trimester (less than 17 weeks)	375 (25.0)	235 (21.5)	140 (34.4)	
2nd trimester (17 weeks to less than 29 weeks)	485 (32.3)	357 (32.6)	128 (31.4)	
3rd trimester (more than 29 weeks)	642 (42.7)	503 (45.9)	139 (34.2)	
Pre-pregnancy BMI *				0.098
Normal (18.5 ≤ BMI < 25.0)	141 (9.4)	99 (9.1)	42 (10.3)	
Underweight (<18.5)	1018 (68.1)	730 (67.0)	288 (70.9)	
Overweight/obese (≥25.0)	336 (22.5)	260 (23.9)	76 (18.7)	
Dairy products *				0.546
Enough	1213 (81.2)	888 (81.6)	325 (80.2)	
Egg *				0.034
Enough	1397 (93.6)	1027 (94.4)	370 (91.4)	
Red meat *				<0.001
Enough	1390 (93.1)	1029 (94.6)	361 (89.1)	
Nut fruits *				0.514
Enough	875 (58.6)	643 (59.2)	232 (57.3)	
Fat (%) (mean ± SD)	35.8 ± 9.0	36.1 ± 9.1	34.9 ± 8.9	0.023
Protein (%) (mean ± SD)	15.3 ± 3.7	15.3 ± 3.7	15.0 ± 3.7	0.147
Carbohydrate (%) (mean ± SD)	49.8 ± 9.8	49.4 ± 9.9	50.8 ± 9.5	0.013
Vitamin D content (g) (median, IQR)	2.8 (7.7)	2.8 (9.6)	2.5 (4.8)	0.031
Vitamin supplements *				<0.001
Vitamin D and/or Calcium	698 (47.7)	560 (52.3)	138 (35.0)	
Sun exposure				0.001
Yes	1046 (69.6)	789 (72.1)	257 (63.1)	
Protective methods for sunshine (mean ± SD)	1.6 ± 1.3	1.6 ± 1.3	1.6 ± 1.3	0.504
Remained indoors during pregnancy				0.018
Yes	228 (15.3)	152 (14.0)	76 (19.0)	
Season of blood draw				0.004
Sunny months	927 (61.7)	700 (63.9)	227 (55.8)	

Abbreviations: BMI, body mass index; IQR, interquartile range; NT$, New Taiwan dollar; SD, standard deviation; VDD, vitamin D deficiency. * Variables containing missingness of ≤0.6%, with the exception of remaining indoors during pregnancy, number of fetuses in this pregnancy, household monthly income, and vitamin supplements, which have 0.9%, 1.1%, 1.9%, and 2.5% missingness, respectively.

**Table 2:** Factors associated with vitamin D deficiency via multiple logistic regression analysis models (*n*= 1502).

Variables	Model 1			Model 2		
	OR	95% CI	*p*	OR	95% CI	*p*
Age	0.96	0.93–0.98	0.005	0.95	0.93–0.98	<0.001
Residential area						
North	1.00					
Central	0.68	0.50–0.94	0.021	0.66	0.48–0.90	0.010
South and east	0.22	0.14–0.33	<0.001	0.20	0.13–0.31	<0.001
Eastern and outlying Islands	0.36	0.25–0.52	<0.001	0.33	0.23–0.47	<0.001
The ordinal of current pregnancy (parity)						
1st child	1.00					
2nd child	0.83	0.62–1.10	0.203			
≥3rd child	0.69	0.42–1.12	0.141			
Gestational age						
1st trimester (less than 17 weeks)	1.00			1.00		
2nd trimester (17 weeks to less than 29 weeks)	0.73	0.52–1.01	0.054	0.72	0.52–0.99	0.046
3rd trimester (more than 29 weeks)	0.61	0.44–0.84	0.002	0.60	0.44–0.83	0.002
Pre-pregnancy BMI						
Normal (18.5 ≤ BMI < 25.0)	1.00					
Underweight (<18.5)	1.04	0.67–1.59	0.850			
Overweight/obese (≥25.0)	0.87	0.63–1.20	0.397			
Egg intake						
Not enough	1.00					
Enough	0.72	0.43–1.23	0.236			
Red meat intake						
Not enough	1.00			1.00		
Enough	0.54	0.34–0.86	0.010	0.50	0.32–0.78	0.002
Fat (%)	0.99	0.98–1.01	0.711			
Vitamin D content						
≤median	1.00			1.00		
>median	0.80	0.62–1.03	0.091	0.78	0.60–1.00	0.057
Vitamin supplements						
No relevant supplements	1.00			1.00		
Vitamin D and/or calcium	0.47	0.36–0.62	<0.001	0.51	0.39–0.66	<0.001
Sun exposure						
No	1.00			1.00		
Yes	0.77	0.59–1.01	0.064	0.75	0.57–0.98	0.034
Remained indoors during pregnancy						
No	1.00			1.00		
Yes	1.33	0.95–1.87	0.089	1.35	0.97–1.88	0.071
Season of blood draw						
Rainy months	1.00					
Sunny months	0.57	0.44–0.75	<0.001	0.59	0.46–0.77	<0.001

Abbreviations: BMI, body mass index; CI, confidence interval; NT$, New Taiwan dollar; OR, odds ratio.

## Data Availability

Not applicable.

## Supplementary Materials

The following supporting information can be downloaded at: https://www.mdpi.com/article/10.3390/nu15051182/s1, Table S1: Spearman’s correlations among the studied variables (*n* = 1502).

## References

[1] Kiely M.E., McCarthy E.K., Hennessy Á. (2021). Iron, iodine and vitamin D deficiencies during pregnancy: Epidemiology, risk factors and developmental impacts. Proc. Nutr. Soc..

[2] Roth D.E., Abrams S.A., Aloia J., Bergeron G., Bourassa M.W., Brown K.H., Calvo M.S., Cashman K.D., Combs G., De-Regil L.M. (2018). Global prevalence and disease burden of vitamin D deficiency: A roadmap for action in low- and middle-income countries. Ann. N. Y. Acad. Sci..

[3] Föcker M., Antel J., Grasemann C., Führer D., Timmesfeld N., Öztürk D., Peters T., Hinney A., Hebebrand J., Libuda L. (2018). Effect of an vitamin D deficiency on depressive symptoms in child and adolescent psychiatric patients—A randomized controlled trial: Study protocol. BMC Psychiatry.

[4] Woon F.C., Chin Y.S., Ismail I.H., Abdul Latiff A.H., Batterham M., Chan Y.M., On Behalf Of The Micos Research Group (2020). Maternal Vitamin D Levels during Late Pregnancy and Risk of Allergic Diseases and Sensitization during the First Year of Life-A Birth Cohort Study. Nutrients.

[5] Balcells M.E., García P., Tiznado C., Villarroel L., Scioscia N., Carvajal C., Zegna-Ratá F., Hernández M., Meza P., González L.F. (2017). Association of vitamin D deficiency, season of the year, and latent tuberculosis infection among household contacts. PLoS ONE.

[6] Ghasemian R., Shamshirian A., Heydari K., Malekan M., Alizadeh-Navaei R., Ebrahimzadeh M.A., Ebrahimi Warkiani M., Jafarpour H., Razavi Bazaz S., Rezaei Shahmirzadi A. (2021). The role of vitamin D in the age of COVID-19: A systematic review and meta-analysis. Int. J. Clin. Pract..

[7] Palacios C., Gonzalez L. (2014). Is vitamin D deficiency a major global public health problem?. J. Steroid Biochem. Mol. Biol..

[8] Heyden E.L., Wimalawansa S.J. (2018). Vitamin D: Effects on human reproduction, pregnancy, and fetal well-being. J. Steroid Biochem. Mol. Biol..

[9] Van der Pligt P., Willcox J., Szymlek-Gay E.A., Murray E., Worsley A., Daly R.M. (2018). Associations of Maternal Vitamin D Deficiency with Pregnancy and Neonatal Complications in Developing Countries: A Systematic Review. Nutrients.

[10] Ames B.N., Grant W.B., Willett W.C. (2021). Does the High Prevalence of Vitamin D Deficiency in African Americans Contribute to Health Disparities?. Nutrients.

[11] Women’s Health By the Numbers: Health Disparities in Pregnancy. https://magazine.medlineplus.gov/article/by-the-numbers-health-disparities-in-pregnancy.

[12] Cashman K.D. (2020). Vitamin D Deficiency: Defining, Prevalence, Causes, and Strategies of Addressing. Calcif. Tissue Int..

[13] Powers J.M., Murphy J.E.J. (2019). Sunlight radiation as a villain and hero: 60 years of illuminating research. Int. J. Radiat. Biol..

[14] Leal A., Corrêa M.P., Holick M.F., Melo E.V., Lazaretti-Castro M. (2021). Sun-induced production of vitamin D(3) throughout 1 year in tropical and subtropical regions: Relationship with latitude, cloudiness, UV-B exposure and solar zenith angle. Photochem. Photobiol. Sci.

[15] Benedik E. (2022). Sources of vitamin D for humans. Int. J. Vitam. Nutr. Res..

[16] Grant W.B., Al Anouti F., Moukayed M. (2020). Targeted 25-hydroxyvitamin D concentration measurements and vitamin D3 supplementation can have important patient and public health benefits. Eur. J. Clin. Nutr..

[17] Pérez-López F.R., Pilz S., Chedraui P. (2020). Vitamin D supplementation during pregnancy: An overview. Curr. Opin. Obstet. Gynecol..

[18] Geography of Taiwan. https://en.wikipedia.org/wiki/Geography_of_Taiwan#Climate.

[19] Pham T.T.M., Huang Y.L., Chao J.C., Chang J.S., Chen Y.C., Wang F.F., Bai C.H. (2021). Plasma 25(OH)D Concentrations and Gestational Diabetes Mellitus among Pregnant Women in Taiwan. Nutrients.

[20] Seamans K.M., Cashman K.D. (2009). Existing and potentially novel functional markers of vitamin D status: A systematic review. Am. J. Clin. Nutr..

[21] Cashman K.D., van den Heuvel E.G., Schoemaker R.J., Prévéraud D.P., Macdonald H.M., Arcot J. (2017). 25-Hydroxyvitamin D as a Biomarker of Vitamin D Status and Its Modeling to Inform Strategies for Prevention of Vitamin D Deficiency within the Population. Adv. Nutr..

[22] Ross A.C., Manson J.E., Abrams S.A., Aloia J.F., Brannon P.M., Clinton S.K., Durazo-Arvizu R.A., Gallagher J.C., Gallo R.L., Jones G. (2011). The 2011 report on dietary reference intakes for calcium and vitamin D from the Institute of Medicine: What clinicians need to know. J. Clin. Endocrinol. Metab..

[23] Sempos C.T., Binkley N. (2020). 25-Hydroxyvitamin D assay standardisation and vitamin D guidelines paralysis. Public Health Nutr..

[24] Holick M.F., Binkley N.C., Bischoff-Ferrari H.A., Gordon C.M., Hanley D.A., Heaney R.P., Murad M.H., Weaver C.M., Endocrine S. (2011). Evaluation, treatment, and prevention of vitamin D deficiency: An Endocrine Society clinical practice guideline. J. Clin. Endocrinol. Metab..

[25] Hien V.T., Lam N.T., Skeaff C.M., Todd J., McLean J.M., Green T.J. (2012). Vitamin D status of pregnant and non-pregnant women of reproductive age living in Hanoi City and the Hai Duong province of Vietnam. Matern. Child Nutr..

[26] Yang C., Jing W., Ge S., Sun W. (2021). Vitamin D status and vitamin D deficiency risk factors among pregnancy of Shanghai in China. BMC Pregnancy Childbirth.

[27] Chuang S.C., Chen H.L., Tseng W.T., Wu I.C., Hsu C.C., Chang H.Y., Chen Y.I., Lee M.M., Liu K., Hsiung C.A. (2016). Circulating 25-hydroxyvitamin D and physical performance in older adults: A nationwide study in Taiwan. Am. J. Clin. Nutr..

[28] Taiwan Geographic Coordinates. https://www.geodatos.net/en/coordinates/taiwan.

[29] Leary P.F., Zamfirova I., Au J., McCracken W.H. (2017). Effect of Latitude on Vitamin D Levels. J. Am. Osteopath Assoc..

[30] Al Zarooni A.A.R., Nagelkerke N., Al Marzouqi F.I., Al Darmaki S.H. (2022). Risk factors for vitamin D deficiency in Abu Dhabi Emirati population. PLoS ONE.

[31] AlFaris N.A., AlKehayez N.M., AlMushawah F.I., AlNaeem A.N., AlAmri N.D., AlMudawah E.S. (2019). Vitamin D Deficiency and Associated Risk Factors in Women from Riyadh, Saudi Arabia. Sci. Rep..

[32] Takaoka N., Nishida K., Sairenchi T., Umesawa M., Noguchi R., Someya K., Kobashi G. (2020). Changes in vitamin D status considering hemodilution factors in Japanese pregnant women according to trimester: A longitudinal survey. PLoS ONE.

[33] Perreault M., Atkinson S.A., Meyre D., Fusch G., Mottola M.F. (2020). Summer Season and Recommended Vitamin D Intake Support Adequate Vitamin D Status throughout Pregnancy in Healthy Canadian Women and Their Newborns. J. Nutr..

[34] Savard C., Bielecki A., Plante A.S., Lemieux S., Gagnon C., Weiler H.A., Morisset A.S. (2021). Longitudinal Assessment of Vitamin D Status across Trimesters of Pregnancy. J. Nutr..

[35] Shen Y., Pu L., Si S., Xin X., Mo M., Shao B., Wu J., Huang M., Wang S., Muyiduli X. (2020). Vitamin D nutrient status during pregnancy and its influencing factors. Clin. Nutr..

[36] Schmid A., Walther B. (2013). Natural Vitamin D Content in Animal Products. Adv. Nutr..

[37] Di Filippo L., De Lorenzo R., Giustina A., Rovere-Querini P., Conte C. (2022). Vitamin D in Osteosarcopenic Obesity. Nutrients.

[38] Gallagher J.C., Yalamanchili V., Smith L.M. (2013). The effect of vitamin D supplementation on serum 25OHD in thin and obese women. J. Steroid Biochem. Mol. Biol..

[39] Savastano S., Barrea L., Savanelli M.C., Nappi F., Di Somma C., Orio F., Colao A. (2017). Low vitamin D status and obesity: Role of nutritionist. Rev. Endocr. Metab. Disord..

[40] Yang Y., Cai Z., Zhang J. (2021). The effect of prepregnancy body mass index on maternal micronutrient status: A meta-analysis. Sci. Rep..

[41] Bodnar L.M., Catov J.M., Roberts J.M., Simhan H.N. (2007). Prepregnancy obesity predicts poor vitamin D status in mothers and their neonates. J. Nutr..

