# Supplementary material for: Effects of Climate, Sun Exposure, and Dietary Intake on Vitamin D Concentrations in Pregnant Women: A Population-Based Study
Source: Nutrients. 2023 Feb 27;15(5):1182. doi: 10.3390/nu15051182 (PMC10005797; doi:10.3390/nu15051182)
Supplement: Supplementary file 1 [file nutrients-15-01182-s001.zip › nutrients-2224259-supplementary.pdf]

Title

Table S1. Spearman's correlations (rho) among the studied variables (n= 1502).

Variables	Age	Living area	Gravidity	Parity	Gestational age	Pre-pregnancy BMI	Egg intake	Cattle meat intake	Fat	Carbohydrate	Vitamin D content	Vitamin supplement	Sun exposure	Stay indoor during pregnancy
Living area	-.16													
Gravidity	.26	.08												
Parity	.23	.12	**.82**											
Gestational age	-.02	.11	-.01	-.00										
Pre-pregnancy BMI	.06	.10	.16	.13	-.00									
Egg intake	.04	-.04	.02	.05	.07	.01								
Cattle meat intake	.02	-.00	.04	.04	.08	-.00	.20							
Fat	.01	.14	-.04	-.03	.05	.08	.00	-.00						
Carbohydrate	-.03	-.05	.03	.03	-.04	-.07	-.02	-.03	**-.89**					
Vitamin D content	.02	.00	-.01	-.01	.03	.02	.08	.00	.10	-.13				
Vitamin supplement	.05	-.02	-.13	-.15	.23	-.00	-.07	.00	.04	-.03	.01			
Sun exposure	.02	.03	.07	.10	.05	.02	.04	.03	-.00	.01	-.00	-.00		
Stay indoor during pregnancy	-.02	-.01	-.02	-.09	-.00	-.03	-.06	-.11	.04	-.01	-.00	-.00	-.06	
Season of blood draw	-.01	-.15	-.08	-.07	-.04	-.04	.01	.02	-.09	.07	.08	.01	.04	-.01
